# Hemostasis for a Post-tonsillectomy Hemorrhage in a Patient With Coronavirus Disease

**DOI:** 10.7759/cureus.39432

**Published:** 2023-05-24

**Authors:** Daisuke Iokura, Tsuyoshi Kojima, Yusuke Okanoue, Shuya Otsuki

**Affiliations:** 1 Department of Otolaryngology, Tenri Hospital, Tenri, JPN; 2 Department of Otolaryngology-Head and Neck Surgery, Graduate School of Medicine, Kyoto University, Kyoto, JPN

**Keywords:** pulmonary embolism (pe), postoperative hemorrhage, post-tonsillectomy hemorrhage, tonsillectomy, coronavirus, covid-19

## Abstract

During the coronavirus disease 2019 (COVID-19) pandemic, otolaryngologists should be careful when performing upper airway surgery because of the risk of aerosol generation.

This paper describes the case of a 23-year-old male who was diagnosed with COVID-19 four days after undergoing tonsillectomy. COVID-19 was complicated by pulmonary thromboembolism, and anticoagulation was administered, which caused postoperative hemorrhage. The patient had to undergo another surgery for the control of hemorrhage, during the infective period of COVID-19.

COVID-19 is sometimes associated with venous embolism, and for postoperative patients, treatment should be carefully considered because of the risk of bleeding. The administration of heparin as an anticoagulant would be preferable because heparin allows for dosage adjustment by measuring activated partial thromboplastin time and also allows the rapid cessation of the anticoagulant effect when the administration is discontinued together with antagonization by protamine administration, even if bleeding occurs. When performing surgery for patients with COVID-19, special care is necessary in order not to spread the infection. Even if the preoperative polymerase chain reaction (PCR) test is negative, the patient may be in the incubation period of COVID-19; therefore, caution should be exercised when performing upper respiratory tract surgery such as tonsillectomy.

## Introduction

Since the coronavirus disease 2019 (COVID-19) epidemic, caused by severe acute respiratory syndrome coronavirus 2 (SARS-CoV-2), started in December 2019, hundreds of millions of people have been infected worldwide. COVID-19 has a predilection for infecting the mucosa of the upper and lower airways [1] and can spread through airborne transmission, which occurs during the transmission of aerosols containing infectious pathogens [2]. Therefore, otolaryngologists should be careful when performing upper airway surgery because of the risk of aerosol generation [3].

SARS-CoV-2 affects the endothelium of capillaries, which leads to coagulation abnormalities. Thus, COVID-19 patients have an increased risk of venous thrombosis, such as pulmonary embolism (PE) [4]. Patients who develop venous thrombosis are typically treated with anticoagulants. However, anticoagulation treatment for postoperative patients requires careful consideration and drug dosage because of the risk of bleeding.

## Case presentation

A 23-year-old male suffered from recurrent tonsillitis six times in a year. The patient visited our hospital, and a tonsillectomy was scheduled. The patient had no other medical history and had not been vaccinated against COVID-19. The patient underwent polymerase chain reaction (PCR) testing twice, initially two days before surgery and on the day of hospitalization, that is, the day before surgery, and the results were negative.

A tonsillectomy was performed uneventfully. Buried tonsils were grasped with forceps and medialized. An electrosurgical device was used to remove the tonsils. Adhesion between the tonsils and surrounding tissue was mild. After tonsil removal, slight bleeding was observed at the upper portion of the left tonsillar bed, and a bipolar device was used for hemostasis. The total bleeding amount was minimal. The surgical operating time was 53 minutes. The size of excised tonsils was 27 mm on the right and 28 mm on the left.

**Figure 1:**
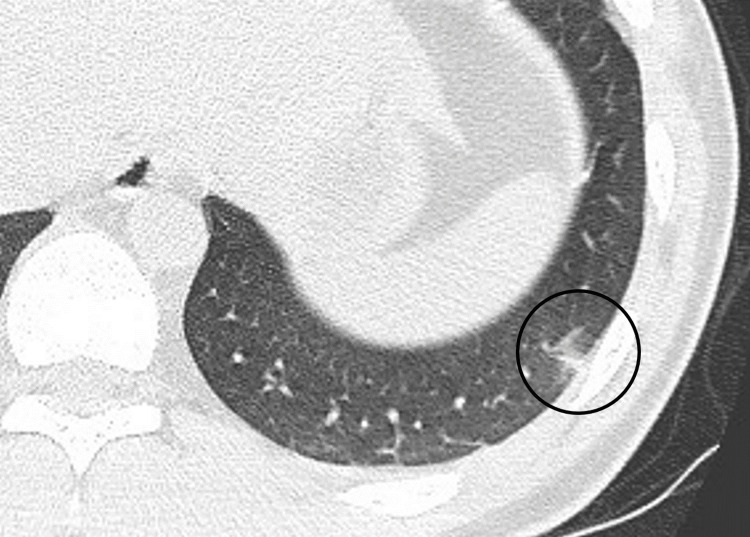
CT revealed a frosted shadow in the lower lobe of the left lung. CT: computed tomography

**Figure 2:**
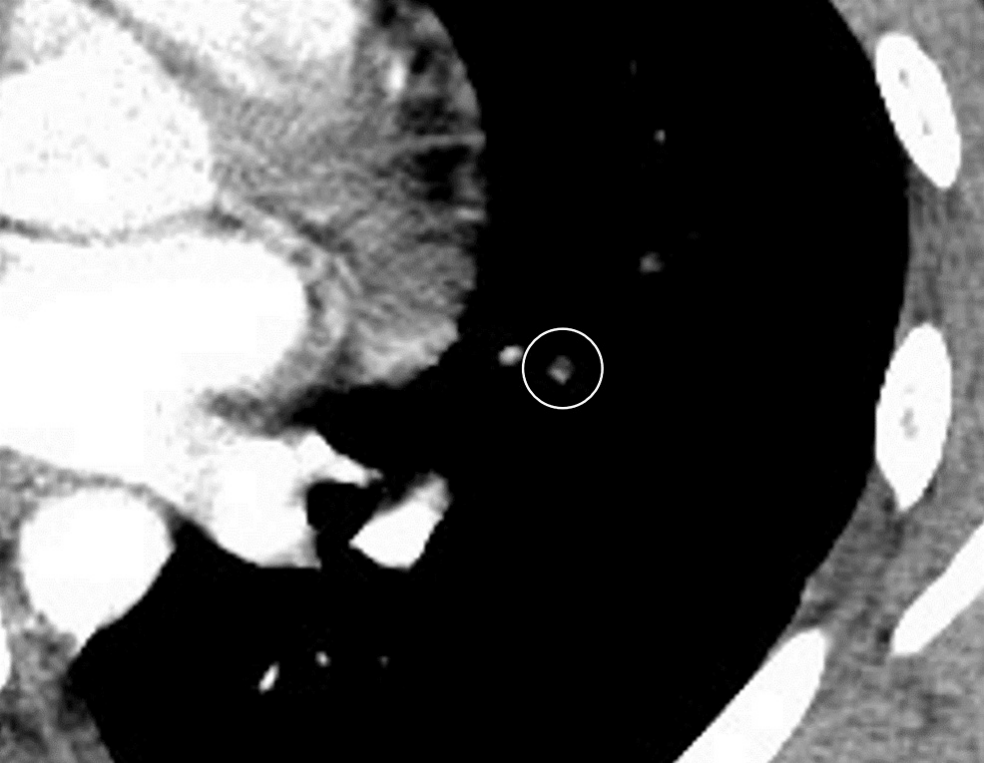
Contrast defects were observed in the left pulmonary artery, and pulmonary thromboembolism (PE) was suspected.

Postoperatively, the tonsil beds were coated in a whitish layer of slough. The patient was scheduled for discharge on the fifth postoperative day, which was a natural course in our hospital. However, on the fourth postoperative day, the patient developed a fever of 38.7℃, cough, and fatigue. The patient had no decrease in oxygenation or blood pressure. A nasopharyngeal swab test for COVID-19 was positive for SARS-CoV-2 RNA. Contrast-enhanced chest computed tomography (CT) revealed a frosted shadow in the lower lobe of the left lung (Figure 1). Contrast defects were observed in the left pulmonary artery, and PE was suspected (Figure 2).

Although the frosted shadow was mild, the patient was considered at risk for severe disease owing to concomitant PE; consequently, an antiviral drug, remdesivir 100 mg, was administered for the treatment of COVID-19. In addition, apixaban 20 mg, a direct oral anticoagulant (DOAC), was initiated for PE.

On the sixth postoperative day, postoperative hemorrhage occurred in the left tonsillar bed, coated in a whitish layer of slough. Small amounts of bleeding (<10 cc) were evident several times. Considering the possibility of uncontrolled bleeding or potential for major hemorrhage, emergency surgery for hemostasis was performed under general anesthesia. Bleeding was evident from the site in the left tonsillar bed where intraoperative hemorrhage had been observed. Minor bleeding was also observed in other sites of both tonsillar beds. A bipolar device was used to stop the bleeding.

To minimize the number of staff in contact with the patient, surgery was performed with two otolaryngologists, one anesthesiologist, and one nurse. All staff wore personal protective equipment (PPE), such as hats, eye shields, N95 masks, gowns, and gloves. The patient was transferred to a negative pressure room near the operating room; the patient was intubated in this room and subsequently transferred to the operating room, as there was no negative pressure operating room available. Staff access to the operating room was kept to a minimum. Postoperatively, the patient was brought back to the negative pressure room and extubated. The staff members removed their PPE and showered after the surgery. Instruments and anesthesia equipment in the operating room were decontaminated using alcohol. Postoperatively, no staff member had contracted COVID-19.

**Figure 3:**
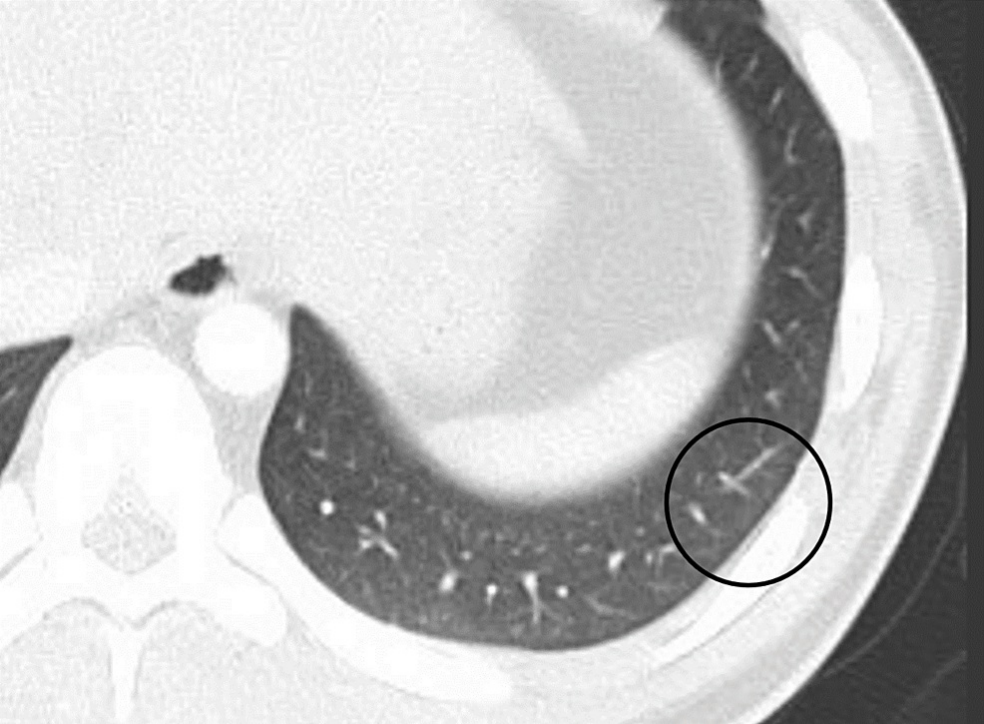
CT scan was performed again, and the frosted shadow of the lower lobe of the left lung had disappeared. CT: computed tomography

**Figure 4:**
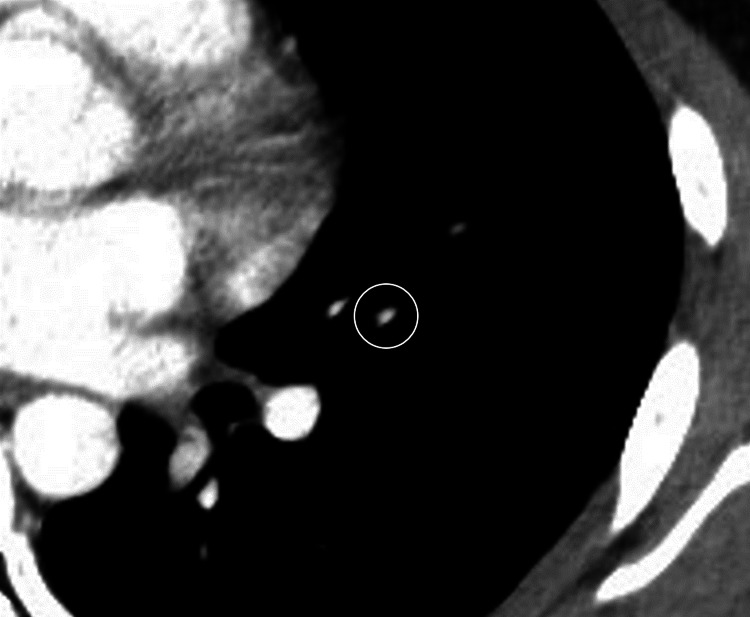
Contrast-enhanced defects in the left pulmonary artery also improved.

Since bleeding was observed from both sides of the tonsillar beds, apixaban medication was thought to be the primary cause of postoperative hemorrhage. Thus, its administration was withdrawn after surgery to enable hemostasis. The following day, a contrast-enhanced chest CT scan was performed again, and the frosted shadow of the lower lobe of the left lung had disappeared (Figure 3). Contrast-enhanced defects in the left pulmonary artery also improved (Figure 4).

Ten days after tonsillectomy and six days after the onset of COVID-19, the fever resolved, and the symptoms of cough and fatigue disappeared. Fourteen days after tonsillectomy and 10 days after the onset of COVID-19, the patient was discharged from our hospital. After discharge, the patient did not rebleed, and the oral mucosal wounds had healed well.

## Discussion

In this case, COVID-19 was complicated by PE, and anticoagulation was administered, which caused a postoperative hemorrhage. The incidence of venous embolism in COVID-19 patients is reported to be 17% (12% for deep vein thrombosis and 7.1% for PE) [5]. Anticoagulation is the mainstay of treatment for acute PE, in patients both with and without COVID-19, to prevent further thrombosis and thromboembolism [6]. The initial treatment options for anticoagulation include unfractionated heparin, low-molecular-weight heparin, fondaparinux, and DOAC. The patient in this case had no decrease in oxygenation or blood pressure, and PE was thought to be at low risk; therefore, the administration of apixaban 20 mg, a DOAC, was initiated. However, a postoperative hemorrhage from the tonsillar bed occurred. Postoperative hemorrhage after tonsillectomy is reported to occur in 0.1%-8.1% of cases [7]. In our hospital, an electrosurgical device is used for tonsillectomy, and surgery for postoperative hemorrhage is rarely required. In this case, the hemorrhage was thought to be caused by apixaban. Anticoagulation in patients with postoperative PE requires the careful consideration of treatment and drug dosage because of the risk of bleeding.

In this case, DOAC was administered as an anticoagulant, but it is possible that the administration of heparin would have been preferable. This is because heparin allows for dosage adjustment while monitoring effectiveness through the measurement of activated partial thromboplastin time (APTT) [8]. The half-life of heparin is short, ranging from 45 to 60 minutes, allowing for the rapid removal of the anticoagulant effect when the administration is discontinued, even if bleeding occurs [8]. In addition, the effect of heparin can be antagonized by protamine administration [8]. Hence, the use of heparin over DOAC is supported in this case.

If possible, surgical procedures for patients with COVID-19 should be delayed owing to the risk of transmitting the infection. It is also recommended that surgery for patients with COVID-19 be delayed by at least seven weeks after infection because of the increased risk of postoperative mortality and pulmonary complications [9]. Many cases have been reported in which patients were asymptomatic preoperatively and were diagnosed with COVID-19 in the perioperative period, resulting in increased postoperative mortality, pulmonary complications, and the infection of the medical staff [10]. Therefore, a screening PCR test is recommended prior to surgery. In the present case, there were no symptoms suspicious for COVID-19 prior to tonsillectomy, and the screening PCR tests were negative; however, on the fourth postoperative day, fever, cough, and malaise appeared, and COVID-19 infection became evident. During the hospitalization period in this case, the Omicron strain was prevalent. The median incubation period for the Omicron strain was 3.1 days, with approximately 20% of patients reported to have an incubation period of five days or longer [11]. The route of infection in this case is not clear, but it is possible that COVID-19 was in the incubation period at the time of PCR testing prior to admission and that it developed after tonsillectomy. When a patient develops fever after surgery, we would normally suspect an infection associated with the surgery or the effects of anesthetics; however, during the COVID-19 pandemic, PCR testing should be performed for the possibility of fever caused by COVID-19.

Tonsillectomy produces aerosols and is associated with a high risk of spreading infection [3]. As in the present case, even if the preoperative PCR is negative, it is possible that the patient may be in the incubation period of COVID-19; therefore, appropriate infection protection should be provided. Moreover, a case has been reported in which tonsillectomy was performed 25 days after COVID-19 infection, and SARS-CoV-2 was detected in the removed tonsil tissue [12]; therefore, we must be careful during tonsillectomy in patients after COVID-19 recovery. Besides wearing appropriate PPE such as surgical masks and eye shields, several other methods have been reported to prevent the spread of infection [13]: Lower power settings of monopolar electrocautery produce fewer aerosols [14], aerosol emissions can be reduced by using a smoke evacuator system [15], and the application of povidone-iodine to the oral cavity and pharynx during surgery may be recommended because it has been reported to inactivate the SARS-CoV-2 virus [16,17]. Therefore, caution should be exercised when performing tonsillectomies during the COVID-19 pandemic.

## Conclusions

COVID-19 is often associated with venous embolism, such as PE, and postoperatively, treatment should carefully consider the risk of bleeding. The administration of heparin as an anticoagulant would be preferable because heparin allows for the adjustment of the dosage by measuring APTT, and it allows the anticoagulant effect to rapidly cease after the discontinuation of administration together with antagonization by protamine administration, even if bleeding occurs.

During the COVID-19 pandemic, a PCR screening test is recommended for patients undergoing surgery. Even if the preoperative PCR test is negative, the patient may be in the incubation period of COVID-19; therefore, caution should be exercised when performing surgery, especially when performing upper respiratory tract surgery such as tonsillectomy.
